# Phosphatase control of cytokine-mediated overproduction of galactose-deficient IgA1, the main autoantigen in IgA nephropathy

**DOI:** 10.1016/j.jaut.2022.102883

**Published:** 2022-08-17

**Authors:** Colin Reily, Terri Rice, David K. Crossman, Dana V. Rizk

**Affiliations:** a Department of Medicine, Division of Nephrology, University of Alabama, Birmingham, USA; b Department of Microbiology, University of Alabama at Birmingham, USA; c Department of Genetics, University of Alabama at Birmingham, Birmingham, AL, USA

## Abstract

IgA nephropathy (IgAN) is an autoimmune disease characterized by the deposition of galactose-deficient IgA1 (Gd-IgA1)-containing immune complexes in the kidneys. Elevated serum levels of Gd-IgA1, the main autoantigen in IgAN, are associated with mucosal infections and poor renal outcome in IgAN patients, but little is known about the activation of IgA1-secreting cells overproducing this autoantigen. We found that in peripheral blood mononuclear cells (PBMCs), cytokine stimulation elevated Gd-IgA1 production in B cells from IgAN patients but not in those from healthy controls (p < 0.01). These results were replicated in immortalized B cells derived from PBMCs of IgAN patients and healthy controls. Using single-cell transcriptomics, we identified subsets of IgA1-secreting cells from IgAN patients, but not from healthy controls, with decreased expression of *C1GALT1* in response to cytokine stimulation. The *C1GALT1-*encoded glycosyltransferase is responsible for addition of galactose to IgA1 *O*-glycans, and its reduced activity is associated with elevated serum levels of Gd-IgA1. These newly identified subsets of IgA1-secreting cells with reduced *C1GALT1* expression exhibited reduced expression of several genes related to cytokine-mediated signaling, including those encoding phosphatases, such as *SOCS1*. siRNA knock-down of *SOCS1*, and the related *SOCS3*, increased Gd-IgA1 production in cells derived from PBMCs of healthy controls, indicating a role of these regulators in abnormal cytokine signaling and Gd-IgA1 overproduction. These results revealed that specific subsets of IgA1-secreting cells may be responsible for autoantigen production in IgAN due to abnormal regulation of cytokine-mediated signaling, a process that may occur in inflammatory responses in IgAN patients.

## 1. Introduction

IgA nephropathy (IgAN) is the most common primary glomerulonephritis worldwide. It is characterized by mesangial deposition of IgA-containing immune complexes with co-deposition of IgG [1-3]. Complement C3 is usually also present [4]. Glomerular IgA immunodeposits in IgAN patients are of the IgA1 subclass and enriched for galactose-deficient *O*-glycoforms (Gd-IgA1) [5,6]. Serum levels of Gd-IgA1 are elevated in IgAN patients [7]. The current understanding of IgAN pathogenesis suggests that circulatory Gd-IgA1 is bound by IgG autoantibodies specific for Gd-IgA1, forming pathogenic immune complexes, some of which deposit in the kidneys triggering a cascade of events leading to renal injury and, in many patients, to kidney failure [8,9]. This hypothesis is supported by evidence that IgG isolated from the kidney immunodeposits is specific for Gd-IgA1 and that Gd-IgA1-IgG immune complexes are found in the circulation of IgAN patients [1,10,11]. However, despite the critical role of Gd-IgA1 in IgAN, the origin of this autoantigen and the characteristics of specific cell population(s) producing Gd-IgA1 remain unknown.

Human IgA is comprised of two subclasses, IgA1 and IgA2; IgA1 contains an extended 18-amino-acid hinge region. Circulatory IgA1 usually contains 3 to 6 core 1 *O*-glycans per hinge region [12]. The *O*-glycan synthesis is initiated by *N*-acetylgalactosamine (GalNAc) transferases (GalNAcTs) that attach GalNAc to some Serine (Ser)/-Threonine (Thr) residues in the IgA1 hinge region [13,14]. This is normally followed by the addition of galactose (Gal), α1,3-linked to GalNAc, by a Core 1 galactosyltransferase (C1GalT1) [14-16]. IgA1 with some glycans lacking Gal is termed Gd-IgA1. Genome wide association studies (GWAS) identified *C1GALT1* SNPs that are associated with elevated Gd-IgA1 serum levels in IgAN patients, highlighting the role of this gene and the encoded enzyme in the autoantigen production and disease pathobiology [17,18]. In addition to genetic components, Gd-IgA1 production is impacted by some cytokines. Prior studies using IgA1-producing cell lines showed that interleukin 4 (IL-4) and IL-6 decrease *C1GALT1* expression and increase Gd-IgA1 production, but only in cells derived from IgAN patients [19]. Stimulation of the IgA1-producing cell lines with single cytokines, IL-6 and leukemia inhibitor factor (LIF) resulted in enhanced and prolonged activation of STAT3 and STAT1 (signal transducer and activator of transcription 1/3), respectively, the likely mechanisms associated with Gd-IgA1 overproduction [20,21]. These studies thus showed that some cytokines can modulate expression of glycosylation enzymes and increase Gd-IgA1 production in IgAN, suggesting possible abnormalities of control mechanisms related to pro-inflammatory cytokine signaling.

It is well accepted that IgA1-secreting cells *in vivo* are exposed to cytokine mixtures and, usually, not to a single cytokine. The *in vivo* conditions that lead to elevated serum levels of Gd-IgA1 are unknown, but can be associated with mucosal infections, as IgAN patients often present with synpharyngitic hematuria [11,22]. This suggests that a cytokine milieu may promote Gd-IgA1 production, leading to formation of new immune complexes that may deposit in the glomeruli and further enhance kidney injury. We have therefore tested a mixture of cytokines (IL-4, IL-6, IL-21, CD40L) and found that it can increase Gd-IgA1 production in peripheral blood mononuclear cells (PBMCs) as well as in immortalized B cells derived from PBMCs of IgAN patients but not in those of healthy controls. In immortalized B cells, our preliminary data showed no significant difference in Gd-IgA1 or IgA1 production between single-cytokine vs. cytokine-mixture stimulation. However, based on our flow cytometry data, there appears to be substantial differences in the activation of some signaling targets, such as pSTAT1/3, between the single vs. multi-cytokine stimulation. This suggests that delineating potential *in vivo* targets relevant to Gd-IgA1 production should include testing with a cytokine mixture methodology.

As Gd-IgA1 constitutes a minority of total circulatory IgA1, we hypothesized that only a subpopulation(s) of IgA1-secreting cells may be responsible for Gd-IgA1 production, both at baseline and during episodes of inflammatory stimuli. To test this hypothesis, we performed single-cell transcriptomic experiments using immortalized IgA1-secreting cells derived from PBMCs of IgAN patients and healthy controls. The data were then analyzed by unsupervised UMAP (Uniform Manifold Approximation and Projection). This analysis revealed unique subpopulations of IgA1-secreting cells with decreased *C1GALT1*-expression in the cells from IgAN donors but not healthy controls after cytokine stimulation. This reduced *C1GALT1* expression thus constitutes a molecular signature of subpopulations producing Gd-IgA1 in response to cytokines. Additionally, pathway analysis of the IgAN-derived cell subpopulations with repressed *C1GALT1* expression showed substantial differences in cytokine-mediated signaling pathways, as well as related phosphatase genes. Gene-specific siRNA knock-down of two phosphatase regulatory proteins identified in Gd-IgA1-secreting subpopulations, SOCS1 and SOCS3 (suppressor of cytokine stimulation 1 and 3), led to Gd-IgA1 overproduction in cells derived from PBMCs of healthy-control due to cytokine stimulation. These results support the hypothesis that subpopulations of IgA1-secreting cells may differentially respond to pro-inflammatory cytokines due to abnormal phosphatase regulation, subsequently leading to overproduction of Gd-IgA1, the main autoantigen in IgAN.

## 2. Materials and methods

### 2.1. Patients

Eligible adult patients (≥18 years of age) with biopsy-proven primary IgAN receiving care at the University of Alabama at Birmingham Nephrology clinic were approached for study participation. Healthy volunteers were recruited from the Birmingham community. Some responded to our study advertisement, others (who had previously participated in our studies) were contacted by our research team if they had indicated their interest in being included in future research opportunities.

All recruited individuals signed an informed consent. The study protocol was reviewed and approved by our institutional review board. The study was conducted in accordance with the principles of the declaration of Helsinki. For the purpose of the data generated in the current manuscript, samples from 5 healthy controls (HC) and 3 IgAN patients were included. In addition, we have over 500 IgAN patient and healthy control donor immortalized B cell lines stored in our biobank which were used for some of the experiments.

Approximately 40 mL of peripheral blood was collected from IgAN and HC donors, and PBMCs were isolated using a gradient centrifugation. Cells were then cultured for 72 h in RPMI 1640 (Gibco), with penicillin/streptomycin (50 u/mL, 50 μg/mL) at 37 °C, 5% CO_2_/95% humidity without and with stimulation with a cytokine mixture (IL-4, IL-6, IL-21, CD40L; 50 ng/mL). For immortalized B cells (using Epstein Barr viral construct [16]), a random subset was selected from previously collected, biobanked samples derived from PBMCs of IgAN patients and HC donors. IgA and Gd-IgA1 concentrations were analyzed in cell-culture media 72 h after cytokine supplementation. Lectin ELISA for Gd-IgA1 used biotin-labeled lectin from *Helix pomatia* (HPA; Sigma Aldrich) [23,24]. Briefly, 100 ng of IgA was loaded in the wells of ELISA plate, incubated, washed, followed by neuraminidase treatment to remove sialic acid. HPA binds to GalNAc but not to GalNAc-Gal or sialylated GalNAc, thus indicating galactose deficiency. A degree of galactose deficiency was then calculated in reference to Gd-IgA1 standard protein [23,24].

### 2.2. Single-cell transcriptomics

To assess transcriptional responses to cytokine mixture in IgA1-producing cells, 10X Genomics single-cell transcriptome 3′ kits were used. The data were compiled using the 10X Genomics Ranger tool. From immortalized B cells derived from PBMCs from 4 healthy controls and 4 patients with IgAN, over 52,000 cells were analyzed, with an average mean read per cell of 44,281 and a median of 2781 genes per cell. Further processing used an open-source R software tool from Dr. Satija’s lab, Seurat V4.01 (https://satijalab.org/seurat/), and a software package from Alteryx for the flexible design of large-scale database analysis (www.alteryx.com). In addition, scaffold gene data (non-contiguous gene sequences not in hg38) from published sources on splice variants of *IGHA1* (immunoglobulin heavy chain A1) that determine secreted (*IGHA1s*) *vs.* membrane-bound (*IGHA1m*) IgA1 isoforms were inserted into the hg38 gene reference database [25]. Based on this approach, we identified 1164 and 1400 IgA1-secreting cells (*IGHA1s)* from healthy-control- and IgAN-derived cells, respectively. These *IGHA1s*-subpopulations were then grouped based on gene co-expression using the non-linear dimension reduction algorithm UMAP. Broad Institute GO biological GSEA pathway analysis was performed on top genes from unique groupings in healthy control (HC) and IgAN UMAP. Differential gene markers for UMAP identified subgroups were identified using Seurat function calls. Analysis of UMAP subgroup gene log2 fold changes within HC and IgAN were performed by comparing a specific subgroup gene expression within either HC or IgAN to all cells in the opposing group (e.g. IgAN group #6 vs all HC cells). Analysis of specific low and high gene expressing subpopulations within the *IGHA1s*-subpopulations were performed with Alteryx by segregating the top 5% and bottom 5% of expressing cells.

### 2.3. siRNA knock-down

OnTargetPlus SmartPool siRNA sequences (Dharmacon) of *SOCS1* and *SOCS3* were used for knock-down. Electroporation was performed using the Amaxa Nucleofector II (Lonza) at setting X-001 with the Amaxa Nucelofector Kit C (Lonza). Immortalized B cells were suspended in electroporation buffer (2 × 106 cells in 100 μL), and siRNA added to a final concentration of 5 nM, followed by electroporation, and incubated in 2 mL of RPMI 1640 for 4 h at 37 °C. Cells were then collected by centrifugation and resuspended in 2 mL of fresh RPMI 1640 media and incubated for 48 h. Medium was replaced, and cell stimulated with cytokine mixture for 72 h, followed by IgA and Gd-IgA1 ELISA analysis, as described above.

### 2.4. Statistical analysis

Statistical analysis of changes in Gd-IgA1 and gene-specific subpopulations in Alteryx were performed using Data Analysis package in Excel and Alteryx, specifically the students t-test (two-tailed paired sample for means). Differential marker analysis performed on single-cell subpopulations employs non-parametric Wilcoxon rank sum test. The Kolmogorov–Smirnov test was used to determine significance for pathway analysis. Differential markers from Seurat v4.0 uses a Bonferroni adjustment for all p-values to yield a p-adj value for individual genes.

## 3. Results

### 3.1. Cytokine stimulation increased Gd-IgA1 production in PBMCs from IgAN patients

**Fig. 1.**
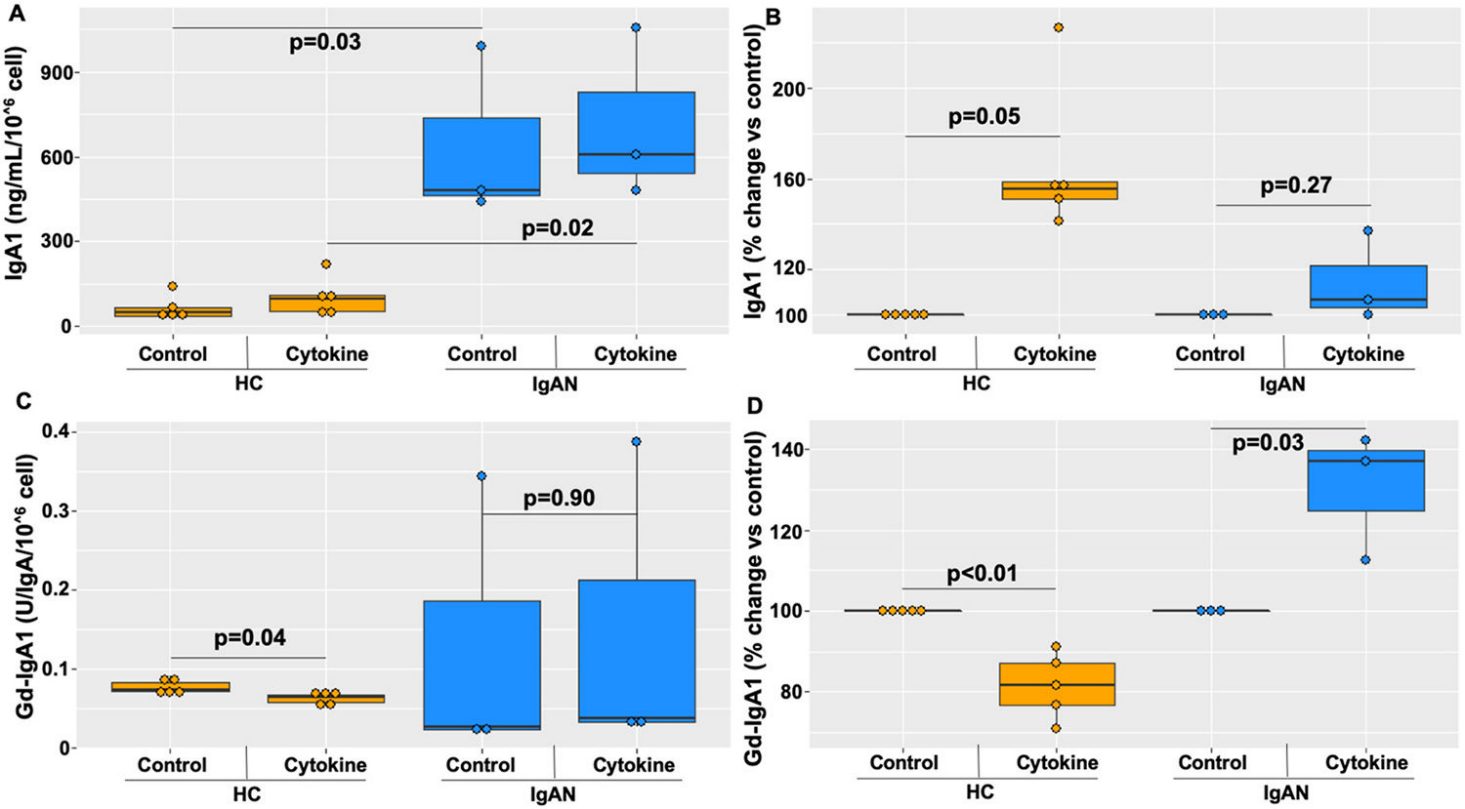
Differential IgA1 and Gd-IgA1 production, with and without cytokine stimulation, by PBMCs from IgAN patients (n = 3) and healthy controls (HC) (n = 5): IgA1 production by the cultured PBMCs was higher in samples from IgAN patients compared to those from HC (p = 0.03) (A). After cytokine stimulation, PBMCs from HC cells increased IgA1 production (p = 0.05) while those from IgAN patients did not (p = 0.27) (B). Although there was no difference in Gd-IgA1 production levels between HC and IgAN PBMCs at baseline (control p = 0.63; cytokine p = 0.45) (C), IgAN-derived PBMCs increased Gd-IgA1 production in response to cytokines (p = 0.03), while HC showed a significant decrease in Gd-IgA1 (p < 0.01) (D).

Cultured PBMCs of IgAN patients produced more IgA1 than those of HC, when normalized to cell number, both at baseline (p = 0.03) and after cytokine stimulation (p = 0.02) (Fig. 1A). No change in IgA1 production was noted after cytokine stimulation within the groups. This difference between IgAN and HC PBMCs could be due to increased number of IgA1-secreting cells in circulation or enhanced production of IgA1. IgA1 production, as a percent change from control, was increased in healthy controls, but not IgAN patients, after cytokine stimulation (Fig. 1B; p = 0.05 and p = 0.27 respectively). Baseline and post-cytokine treatmemt Gd-IgA1 production was not different between the cells from IgAN patients vs. healthy controls, when normalized to cell number. There was significant decrease in Gd-IgA1 production within the HC group after cytokine stimulation (p = 0.04), but no change within the IgAN PBMC group (p = 0.90) (Fig. 1C). However, cytokines led to a relative increase in Gd-IgA1 production by PBMCs but only within the IgAN patient group (Fig. 1D; p = 0.03), with a relative decrease in the HC derived cell group (Fig. 1D; p < 0.01).

### 3.2. Cytokine stimulation increased Gd-IgA1 production by immortalized B cells from IgAN patients

**Fig. 2.**
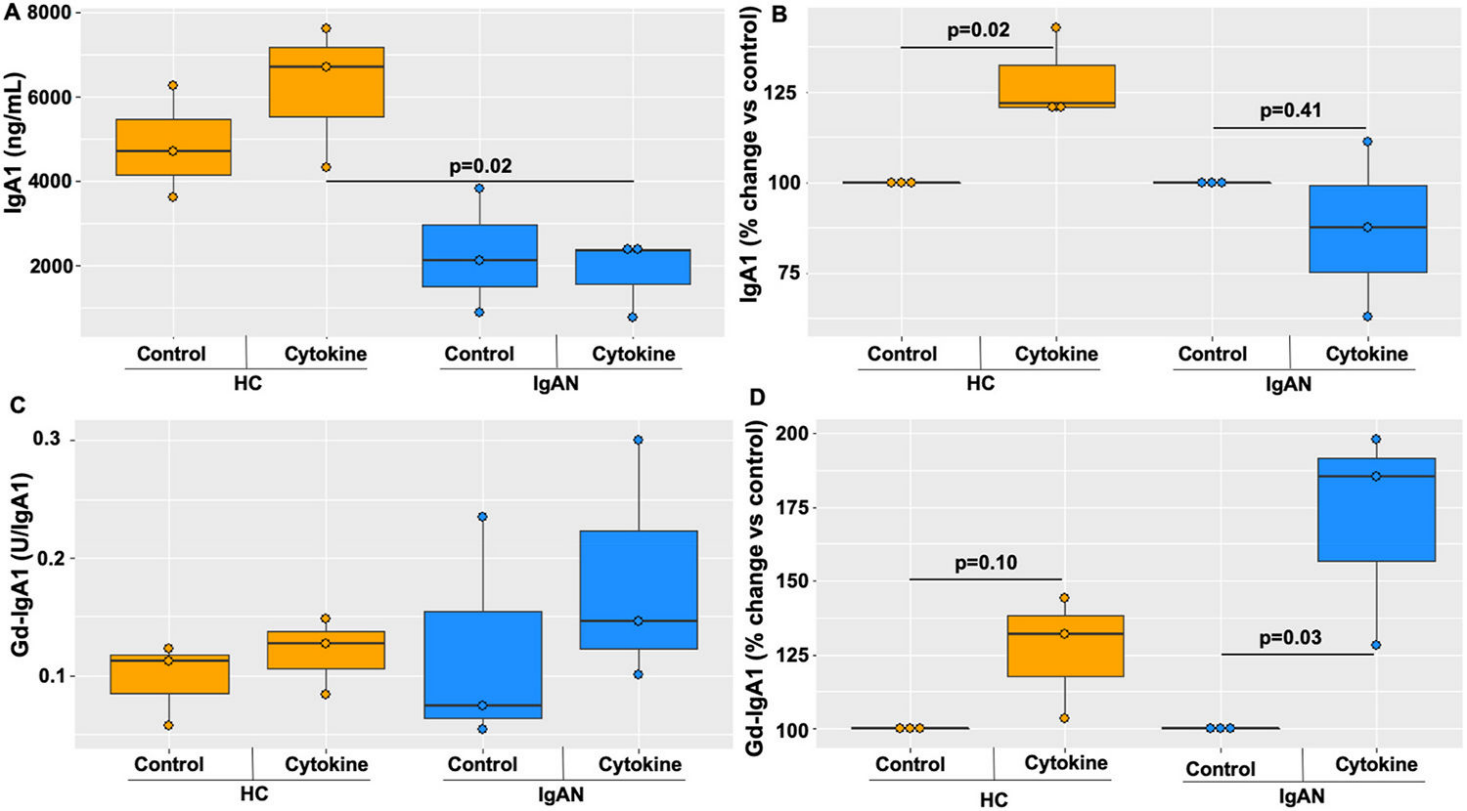
IgA1 and Gd-IgA1 production after cytokine stimulation differs between immortalized B cells from IgAN patients (n = 3) and healthy controls (n = 3): IgA1 production by immortalized B cells was lower in samples from IgAN patients compared to those of healthy controls (HC) (p = 0.02) (A). HC had a higher increase in IgA1 production after cytokine stimulation compared to IgAN-derived B cells (B). There was no difference in baseline Gd-IgA1 production between HC and IgAN PBMCs (C), but IgAN-derived cells had higher increase in Gd-IgA1 production in response to cytokines compared to HC (p = 0.03), with no statistically significant change for HC (p = 0.10) (D).

Cytokine stimulation increased IgA1 production by immortalized B cells derived from PBMCs of HC compared to those of IgAN patients (Fig. 2A; p = 0.02), with no change within groups (Fig. 2A). IgA1-production, as a percent change to control, found no differences between the two groups (Fig. 2B), but did show a significant increase within the HC group, and no change within the IgAN group. Baseline Gd-IgA1 production was not different between IgAN- vs HC-derived cells, and no difference within groups (Fig. 2C). Gd-IgA1 production, as a percent change from control, showed increased Gd-IgA1 production by cells within the IgAN patient group (Fig. 2D; p = 0.03), but not those within the HC group (Fig. 2D; p = 0.10). We did not need to normalize for cell number since all immortalized B cell experiments were plated at the same cell concentration, however, this does not account for potential differences between groups in the percent of IgA1^+^ cells since we are using mixtures of B cells that contain all possible classes. We did not see a decrease in Gd-IgA1 production within the immortalized HC B cell group like we did in the PBMC data, which could be due to needing a higher number of replicates since immortalization also just captures a small percentage of all the B cells found in a PBMC sample.

### 3.3. UMAP of single-cell transcriptomics of immortalized IGHA1sB cells after cytokine stimulation

**Fig. 3.**
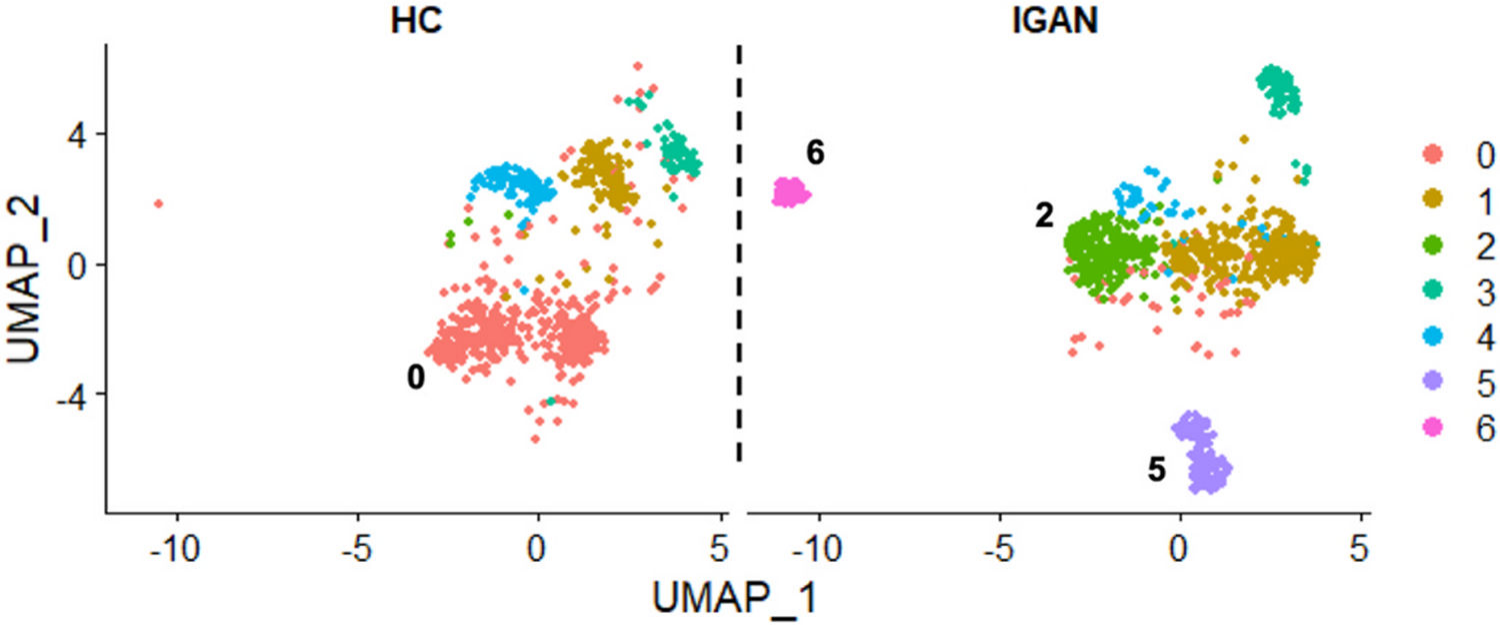
Single-cell transcriptomic UMAP of HC and IgAN immortalized B cells after cytokine stimulation: Immortalized B cells from HC (n = 4) and IgAN (n = 4) donors were stimulated with cytokines for 20 min, and then subjected to single-cell transcriptomic analysis. Seurat v4.0 was used to normalize data, *IGHA1s* subpopulations were grouped, and UMAP was used to find common and distinct populations between HC and IgAN stimulated samples positive for IgA1-secreted transcript. The numbers in the graph indicate populations unique for HC (0) and IgAN (2, 5, 6) samples.

Immortalized B cells from IgAN patients and healthy control donors were stimulated with cytokines and differential gene expression was analyzed by single-cell transcriptomics. Unsupervised UMAP analysis of immortalized *IGHA1s* + cells after cytokine stimulation revealed several different cell populations, some common and others unique to either HC or IgAN patients (Fig. 3). This analysis revealed that there were substantial differences within IgA1-secreting cells, and that IgAN-derived cells had unique populations (#2, #5, #6) compared to those from healthy controls (#0).

### 3.4. Pathway analysis of unique subgroups in IGHA1s cells from IgAN and healthy control donors

Pathway analysis using statistically significant genes of the subpopulations from the UMAP separation after cytokine stimulation found common and unique pathways within the IgAN-patient and healthy-control groups. Table 1 lists for each group the unique pathways that were statistically significantly different. While we suspected that intracellular signaling related to cytokine stimulation would be important, we did not anticipate the prevalence of MAPK signaling within the IgAN groups. The Immune System Development pathway had the highest gene ratio number (k/K) of all the pathways, suggesting significant immunity-specific processes may be altered (Table 1). These results together illustrate substantial differences in cytokine-mediated signaling in cells derived from PBMCs of IgAN patients vs. healthy controls.

### 3.5. Specific glycosyltransferases and phosphatases differentially expressed in IGHA1s subpopulations from IgAN patients vs. healthy controls

**Fig. 4.**
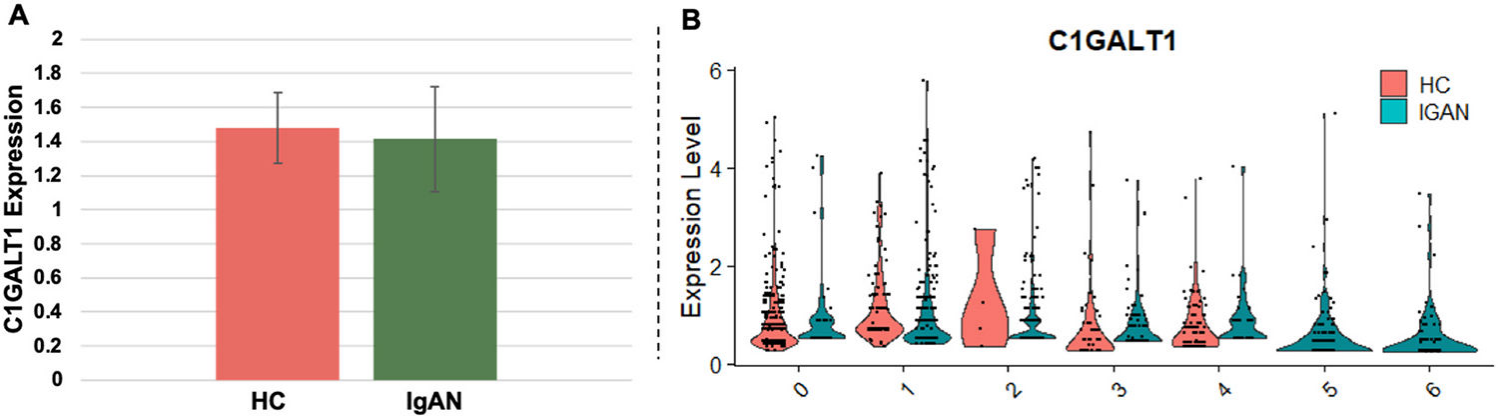
Differential regulation of *C1GALT1* in *IGHA1s* subpopulations: Immortalized B cells from HC (n = 4) and IgAN (n = 4) donors were stimulated with cytokines for 20 min, and then subjected to single-cell transcriptomic analysis. IgA1-secreting cell (*IGHA1s* + cells) subpopulations were identified using UMAP (Fig. 3), followed by differential gene marker analysis to determine differences between groups. (A) Average *C1GALT1* expression between all subgroups in HC- and IgAN-derived B cells with cytokine stimulation, and (B) a breakdown of *C1GALT1* expression for each subpopulation.

The gene critical for Gd-IgA1 production, *C1GALT1*, was significantly downregulated in two of the three IgAN-unique subpopulations (5, 6), but not in any of the HC groups (Table 2, Fig. 4A and B). This suggests that these IgAN-unique cell populations may be producing substantial amounts of Gd-IgA1. Additionally, decreased expression in a sialyltransferase (*ST6GALNAC3*) was observed in the IgAN-unique subpopulations (2, 5, 6), but increased in the HC-unique group (0) (Table 2). Sialyltransferases have been implicated in Gd-IgA1 production previously, such as *ST6GALNAC2*; however, this is the first time *ST6GALNAC3* has been reported to be associated with Gd-IgA1 production [19]. Expression of *GALNT12*, a GalNAc transferase recently reported to be associated with Gd-IgA1 production [26], was also decreased in the HC-unique group (0), and increased in two of the three IgAN-unique groups (5, 6). *SOCS1*, encoding a phosphatase and one of the primary regulators of STAT1 activation, was downregulated in one IgAN-unique group (6). *SOCS3*, encoding a significant regulator of STAT3, was increased in the HC-unique group (0), but no significant changes were found in any of the IgAN-unique groups (Table 2).

### 3.6. B cells from IgAN donors with lowest C1GALT1 expression had highest expression of IGHA1s after cytokine stimulation

**Fig. 5.**
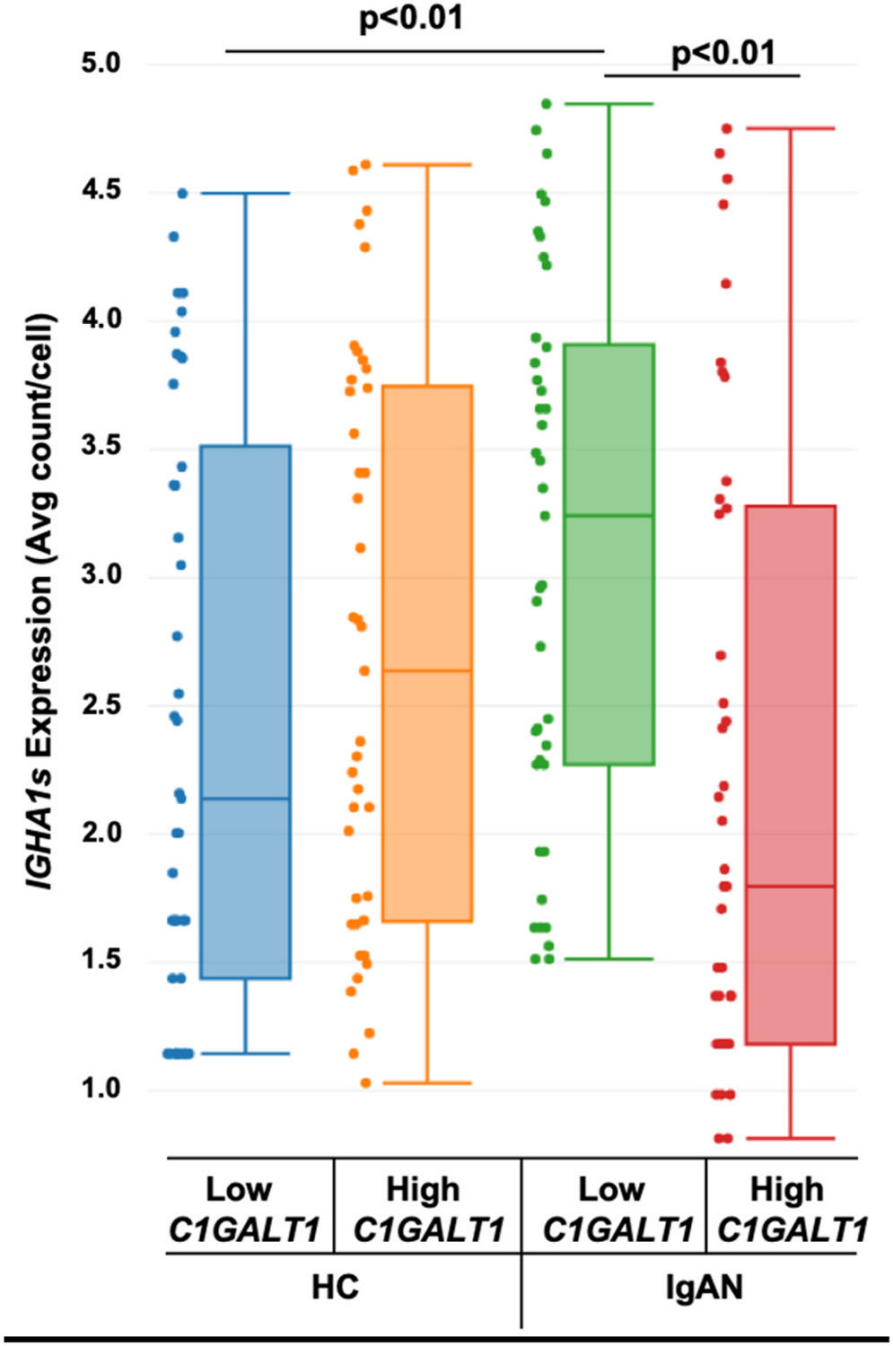
High expression of *IGHA1s* observed in low *C1GALT1*-expressing subpopulations in IgAN-derived cells: Immortalized B cells from HC (n = 4) and IgAN (n = 4) donors were stimulated with cytokines for 20 min, and then subjected to single-cell transcriptomic analysis. *IGHA1s*-expressing cells after cytokine stimulation were separated into low and high *C1GALT1*-expressing groups and assessed for levels of mRNA for IgA1-secreted isoform (*IGHA1s*).

Immortalized B cells stimulated for 20 min with cytokines, were analyzed by single-cell transcriptomics, and separated into high and low *C1GALT1* subpopulations. *IGHA1s*-expression levels were then determined for these groups. In IgAN-derived cells, *IGHA1s*-expression was higher in the low-expressing *C1GALT1* group compared to the high-expressing *C1GALT1* group (p < 0.01) and compared to the low-expressing healthy control group (p < 0.01) (Fig. 5). This observation indicates that cytokine-induced elevated secretion of IgA1 in IgAN patients may occur predominantly in the subpopulations with the lowest expression of *C1GALT1*.

### 3.7. Low expression of C1GALT1 in low-expressing SOCS1 subpopulations in IgAN IGHA1s B cells

**Fig. 6.**
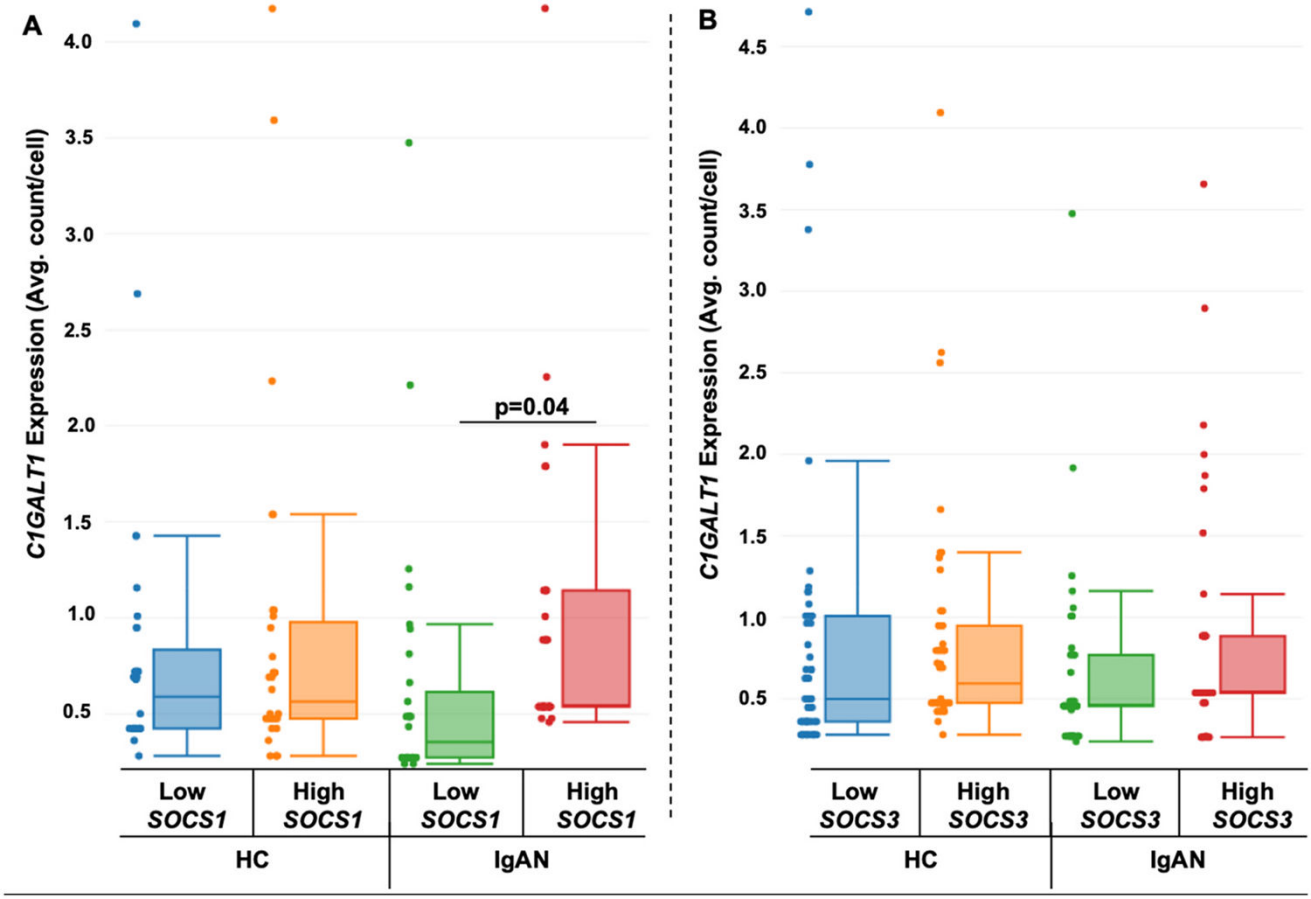
Lower expression of *C1GALT1* occurs in low-expressing *SOCS1* subpopulations from IgAN donors but not HC donors: Immortalized B cells from HC (n = 4) and IgAN (n = 4) donors were stimulated with cytokines for 20 min, and then subjected to single-cell transcriptomic analysis. *IGHA1s*-expressing cells after cytokine stimulation were separated into low and high *SOCS1*-expressing (A) and *SOCS3*-expressing (B) groups and assessed for levels of *C1GALT1*.

Immortalized *IGHA1s* B cells after cytokine stimulation were grouped into low- and high-expressing *SOCS1* and *SOCS3* groups. We found significantly lower *C1GALT1* expression in the low *SOCS1* group compared to high *SOCS1*-expressing group, but only in the IgAN-derived cells (p = 0.04) (Fig. 6A). This difference in *C1GALT1* expression was not seen in the *SOCS3* low- and high-expressing subpopulations (Fig. 6B).

### 3.8. Increased expression of IGHA1s in low SOCS1 and low SOCS3 groups after cytokine stimulation

**Fig. 7.**
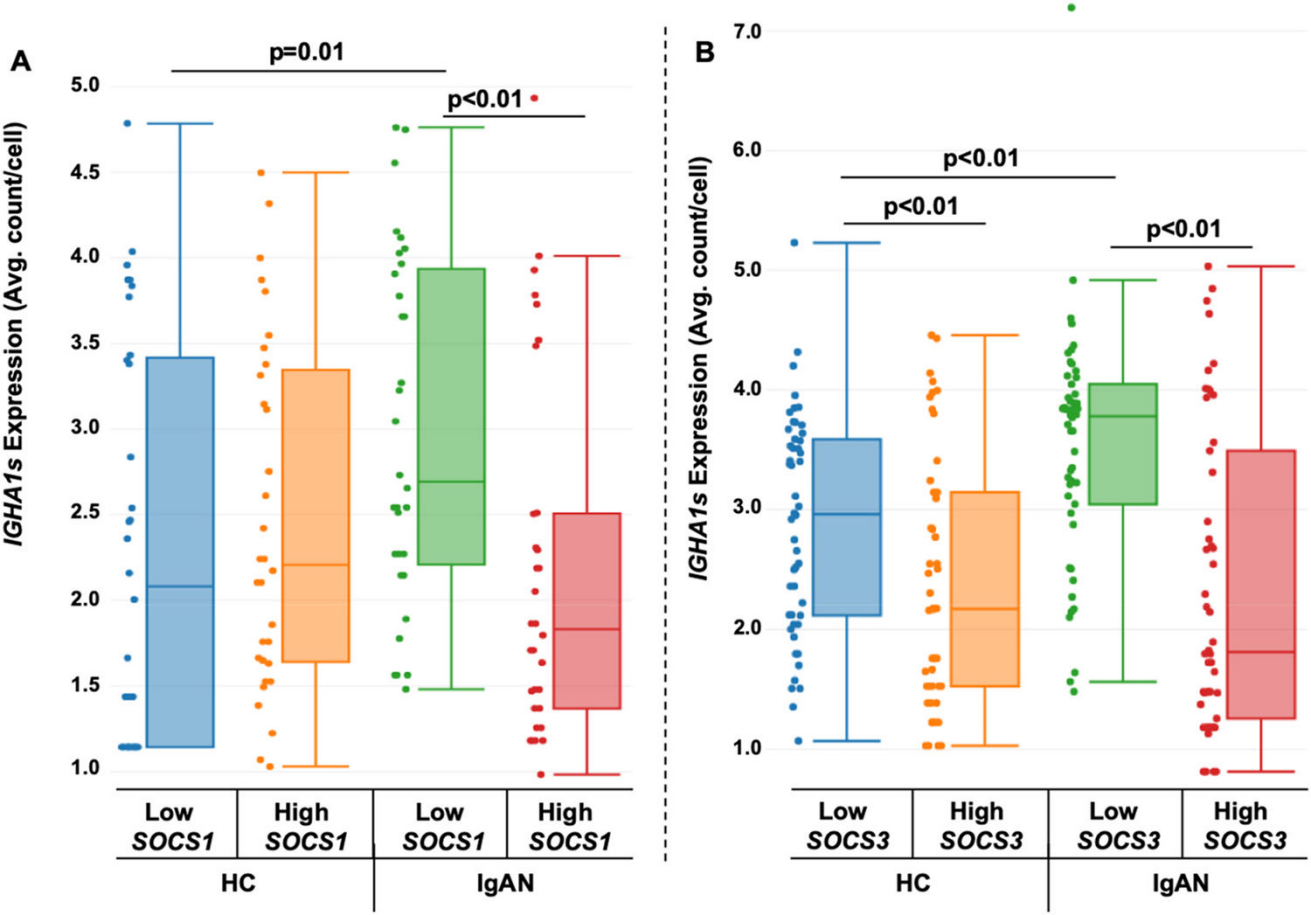
Higher expression of *IGHA1s* in low-expressing *SOCS1* and *SOCS3* subpopulations from IgAN donors compared to HC donors: Immortalized B cells from HC (n = 4) and IgAN (n = 4) donors were stimulated with cytokines for 20 min, and then subjected to single-cell transcriptomic analysis. *IGHA1s*-expressing cells after cytokine stimulation were separated into low and high *SOCS1*-expressing (A) and *SOCS3*-expressing (B) groups and assessed for levels of *IGHA1s*.

To assess the potential connection of phosphatases in IgA1-secretion, we grouped *IGHA1s*-positive cells into low and high *SOCS1* and *SOCS3*-expressing cell subpopulations. Expression levels of *IGHA1s* were higher in low-expressing *SOCS1* group in IgAN-derived cells compared to the high *SOCS1* group (p < 0.01) and the low *SOCS1* healthy-control-derived cells (p = 0.01) (Fig. 7A). Additionally, expression of *IGHA1s* was higher in the low-expressing *SOCS3* group from IgAN-derived cells compared to the high *SOCS3* group (p < 0.01) and to the low-expressing *SOCS3* group from healthy-control-derived cells (p < 0.01) (Fig. 7B).

### 3.9. Increased Gd-IgA1 production in healthy-control cells after siRNA-mediated gene-specific knock-down of SOCS1 and SOCS3

**Fig. 8.**
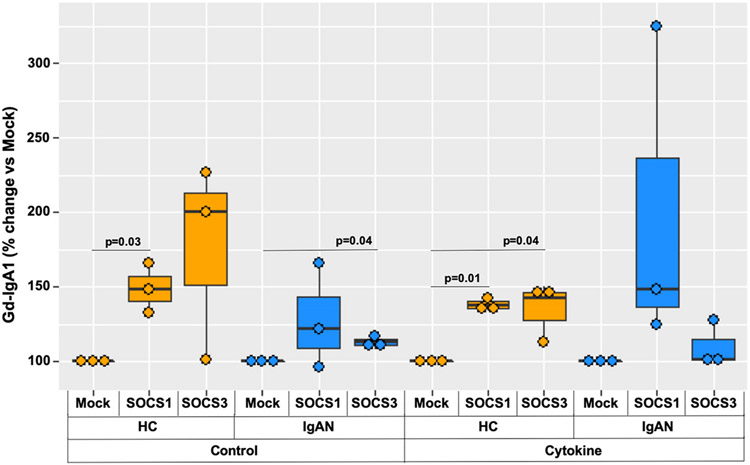
siRNA gene-specific knock-down of *SOCS1* and *SOCS3* increased Gd-IgA1 production: siRNA knock-down of *SOCS1* and *SOCS3* increased Gd-IgA1 production in cytokine-stimulated B cells from HC donors. Knockdown of *SOCS1* in HC-derived B cells after siRNA-mediated knock-down of *SOCS3* in IgAN-derived B cells also increased Gd-IgA1 production without cytokine stimulation.

Expression of *SOCS1* and *SOCS3* was altered using siRNA knockdown in immortalized B cells from HC and IgAN donors, stimulated with cytokines and Gd-IgA1 production was measured. We found increased Gd-IgA1 production after *SOCS1* knock-down in healthy-control cells without stimulation (p = 0.03), and a trend to increased Gd-IgA1 production in the *SOCS3* knockdown. For IgAN-derived cells, we saw a significant increase in Gd-IgA1 without stimulation in the *SOCS3* knock-down (p = 0.04), with a trend to higher Gd-IgA1 production after *SOCS1* siRNA knock-down. After stimulation with cytokines, both the *SOCS1* (p = 0.01) and *SOCS3* (p = 0.04) knock-down increased Gd-IgA1 production in the healthy-control-derived cells, with no significant increase seen in the IgAN-derived cells (Fig. 8).

## 4. Discussion

The role of the autoantigen, Gd-IgA1, in the pathobiology of IgAN has been widely understood as necessary but not sufficient for the development of this disease [14]. However, the origins and mechanisms that mediate the overproduction of Gd-IgA1 during states of disease activity are poorly understood. The correlation between autoantigen production and inflammation can be seen in IgAN patients who present with synpharyngitic hematuria with concurrent elevated levels of Gd-IgA1 and its circulating immune complexes [22,27]. Furthermore, GWAS have supported this connection with inflammation, based on discovery of IgAN-risk-associated genetic alleles, i.e., SNPs, such as those in *HORMAD2* locus with genes encoding also LIF and OSM (oncostatin M) [28]. Moreover, several studies revealed enhanced Gd-IgA1 production in response to IL-6 in IgAN-derived cells, a process involving aberrant signaling leading to reduced expression of *C1GALT1* [19]. Here we show that abnormal responses to cytokines are not limited to single-cytokine exposures, and thus may not be due to only one abnormal proinflammatory pathway (Figs. 1 and 2). Our findings highlight the importance of experimenting with *in vivo* mimicking conditions where IgA1+ B cells are exposed to a multi-cytokine milieu.

Gd-IgA1 in the circulation, mostly bound in immune complexes, is predominantly in the polymeric form, i.e., the molecular form thought to be produced mainly by plasma cells in mucosal tissues [16]. Polymeric IgA1 constitutes only ~10% of the total circulatory IgA1 [29] and Gd-IgA1 in immune complexes represents <1% of total serum IgA1. Thus, we hypothesize that only a small fraction of IgA1-secreting cells is responsible for production of polymeric Gd-IgA1. Single-cell transcriptomic data revealed, for the first time, several unique subpopulations of *IGHA1s*-expressing cells derived from PBMCs of IgAN patients. These cells exhibited reduced *C1GALT1* expression after cytokine stimulation (Table 2, Fig. 4). Of note, the comparison of healthy-control-derived cells before and after cytokine stimulation showed no difference in *C1GALT1* expression, whereas the same pairwise analysis of IgAN-derived samples showed changes in *C1GALT1* expression after cytokine exposure. (S.Tables 1 and 2, S.Figs. 1 and 2). Another glycosyltransferase gene, ST6GALNAC2, has been reported to be overexpressed in immortalized B cells from IgAN patients after cytokine stimulation. However, we have not observed differences among cell subpopulations in our analyses [19]. Conversely, we found that ST6GALNAC3 was decreased in IgAN subpopulations, compared to a relative increase in HC supopulations (Table 2). This may be important in the disease pathogenesis because the Gd-IgA1 in circulating Gd-IgA1-IgG immune complexes lacks a sialic acid on the terminal GalNAc, thus allowing for the binding of *anti*-Gd-IgA1 IgG autoantibodies [10].

The IgAN-unique subpopulations (Fig. 3) had altered cell-signaling pathways (Table 1), as well as differential phosphatase expression of *SOCS1* and *SOCS3* compared to healthy control cells after cytokine stimulation (Table 2). To determine if these specific phosphatases correlated with changes in *C1GALT1*, we separated data for cells with low vs. high *C1GALT1*-expression, and found that low *SOCS1*-expressing subpopulations in IgAN-derived *IGHA1s* cells had decreased expression of *C1GALT1*. Similar analyses were performed for low vs. high expression *SOCS3* groups, but no such correlation was found (Fig. 6). These low *SOCS1*-expressing cells had higher expression of *IGHA1s* and lower expression of *C1GALT1*, indicating a potential for overproduction of Gd-IgA1 (Fig. 7). This is supported by the knockdown of *SOCS1* and *SOCS3* data showing increased Gd-IgA1-production responses to cytokine stimulation in healthy-control-derived cells (Fig. 8). Additionally, other phosphatases, such as *PTPN2* and *PTPN6* (S.Table 3), were differentially regulated in IgAN-unique cells compared to healthy-control-derived cells after cytokine stimulation. These changes in expression of phosphatases may be responsible for abnormal responses to pro-inflammatory processes, as indicated by decreased *NFKBIA* (NF-kappa-B inhibitor A) expression in IgAN-unique groups compared to healthy-control-derived groups (S. Table 3). We pose that multiple phosphatases may act in concert and mediate abnormal cytokine-induced responses that lead to reduced *C1GALT1* expression and, thus, overproduction of Gd-IgA1 in IgAN.

In conclusion, we discovered unique IgA1-secreting cells with differential responses to cytokine stimulation, and showed that some IgAN-unique cell types exhibited reduced expression of *C1GALT1*, leading to Gd-IgA1 overproduction. Our data revealed that abnormal phosphatase regulation is involved in cytokine-mediated autoantigen overproduction. Moreover, a minority of IgA1-secreting cells may be responsible for Gd-IgA1 overproduction. We do not know if the unique IgAN subpopulations (2,5,6) are downstream from a more “normal” subset that occurs due to cytokine stimulation, or if they represent a critical starting subpopulation in IgAN patients. We anticipate that assessing these subpopulations for Gd-IgA1 production will require a combination of cell-surface phenotyping and single-cell transcriptomics of PBMCs in IgAN patients. These findings suggest a possibility of a direct targeting of pro-inflammatoiy regulatory pathways in Gd-IgA1-producing cells and their subsets. The evolution of single-cell transcriptomics and direct antigen-targeting technologies will enable a more details analyses of IgA1^+^ cells to further elucidate inflammatory drivers of dysregulated glycosylation and autoantigen production in IgAN.

## Supplementary Material

Supplemental.Data.Reily.IgAN.phosphatase.2022

## Figures and Tables

**Table 1:** GSEA GO biological pathway analysis from single-cell transcriptomic analysis of cytokine-stimulated IgA1-secreting cells: Immortalized B cells from HC (n = 4) and IgAN (n = 4) donors were stimulated with cytokines for 20 min, and then subjected to single-cell transcriptomic analysis. Cells were grouped based on *IGHA1s*-expression (IgA1-secreting cells), followed by UMAP analysis. GSEA GO biological pathway analysis was performed on the top 200 significant genes from each unique subpopulation (HC: 0, IgAN: 2, 5, 6). Pathways unique to IgAN or HC subgroups are listed. *FDR (false discovery rate*).

Pathway	Genes in pathway	Gene Ratio (k/K)	FDR	Group
**Pathways unique to only IgAN groups**				
MAPK Cascade	65	0.0709	1.5 × 10^−10^	2, 5, 6
Regulation of MAPK Cascade	52	0.0736	5.54 × 10^−10^	5
Immune System Development	77	0.0779	1.19 × 10^−15^	6
Regulation of intracellular signal transduction	99	0.0557	4.41 × 10^−11^	2, 5, 6
Regulation of protein phosphorylation	76	0.0627	9.95 × 10^−11^	2, 6
Response to Cytokine	76	0.0640	6.32 × 10^−12^	2
**Pathways unique to only HC group**				
Cell Activation	87	0.0595	5.59 × 10^−12^	0

**Table 2:** Differential regulation of glycosyltransferase and phosphatase genes in *IGHA1s* subpopulations: Immortalized B cells from HC (n = 4) and IgAN (n = 4) donors were stimulated with cytokines for 20 min, and then subjected to single-cell transcriptomic analysis. IgA1-secreting cell (*IGHA1s*^+^ cells) subpopulations were identified using UMAP (Fig. 3), followed by differential gene marker analysis to determine differences between groups. Listed are some glycosyltransferase and phosphatase genes found to be differentially regulated between IgA1-secreting groups in HC and IgAN. ND = genes with no differential expression from other groups. LFC = log2 fold change.

Select glycosyltransferases and phosphatases in *IGHA1s* subpopulations										
	C1GALT1		ST6GALNAC3		GALNT12		SOCS3		SOCS1	
Group	LFC	p-adj	LFC	p-adj	LFC	p-adj	LFC	p-adj	LFC	p-adj
**0**	0.31	1	1.36	1.18E-86	−1.25	1.63E-88	0.38	1.53E-25	ND	ND
**1**	0.49	2.31E-12	−0.35	2.48E-04	0.53	3.49E-3	ND	ND	ND	ND
**2**	−0.33	1	−1.31	2.33E-31	ND	ND	ND	ND	ND	ND
**3**	−0.35	1	ND	ND	ND	ND	−0.80	9.99E-08	ND	ND
**4**	ND	ND	ND	ND	−0.64	6.83E-05	−0.55	1.67E-08	−0.55	1
**5**	−0.49	3.94E-12	−1.28	1.59E-16	0.77	2.39E-34	ND	ND	ND	ND
**6**	−0.88	3.08E-05	−1.24	4.52E-06	0.73	0.28	−1.13	0.75	−1.08	0.05

## Data Availability

Data will be made available on request.

## References

[1] Rizk DV, Saha MK, Hall S, Novak L, Brown R, Huang ZQ, Fatima H, Julian BA, Novak J, Glomerular immunodeposits of patients with IgA nephropathy are enriched for IgG autoantibodies specific for galactose-deficient IgA1, J. Am. Soc. Nephrol 30 (2019) 2017–2026, 10.1681/ASN.2018111156. 31444275 PMC6779349

[2] Tomino Y, Endoh M, Nomoto Y, Sakai H, Double immunofluorescence studies of immunoglobulins, complement components and their control proteins in patients with IgA nephropathy, Acta Pathol. Jpn 32 (1982) 251–256. 6211893 10.1111/j.1440-1827.1982.tb02046.x

[3] Berger J, Hinglais N, [Intercapillary deposits of IgA-IgG], J. Urol. Nephrol 74 (1968) 694–695. 4180586

[4] Rizk DV, Maillard N, Julian BA, Knoppova B, Green TJ, Novak J, Wyatt RJ, The emerging role of complement proteins as a target for therapy of IgA nephropathy, Front. Immunol 10 (2019) 504, 10.3389/fimmu.2019.00504. 30941137 PMC6433978

[5] Hiki Y, Odani H, Takahashi M, Yasuda Y, Nishimoto A, Iwase H, Shinzato T, Kobayashi Y, Maeda K, Mass spectrometry proves under-*O*-glycosylation of glomerular IgA1 in IgA nephropathy, Kidney Int. 59 (2001) 1077–1085, 10.1046/j.1523-1755.2001.0590031077.x. 11231363

[6] Allen AC, Bailey EM, Brenchley PE, Buck KS, Barratt J, Feehally J, Mesangial IgA1 in IgA nephropathy exhibits aberrant *O*-glycosylation: observations in three patients, Kidney Int. 60 (2001) 969–973, 10.1046/j.1523-1755.2001.060003969.x. 11532091

[7] Moldoveanu Z, Wyatt RJ, Lee JY, Tomana M, Julian BA, Mestecky J, Huang WQ, Anreddy SR, Hall S, Hastings MC, Lau KK, Cook WJ, Novak J, Patients with IgA nephropathy have increased serum galactose-deficient IgA1 levels, Kidney Int. 71 (2007) 1148–1154, 10.1038/sj.ki.5002185. 17342176

[8] Novak J, Julian BA, Mestecky J, Renfrow MB, Glycosylation of IgA1 and pathogenesis of IgA nephropathy, Semin. Immunopathol 34 (2012) 365–382, 10.1007/s00281-012-0306-z. 22434325

[9] Suzuki H, Kiryluk K, Novak J, Moldoveanu Z, Herr AB, Renfrow MB, Wyatt RJ, Scolari F, Mestecky J, Gharavi AG, Julian BA, The pathophysiology of IgA nephropathy, J. Am. Soc. Nephrol 22 (2011) 1795–1803, 10.1681/ASN.2011050464. 21949093 PMC3892742

[10] Tomana M, Novak J, Julian BA, Matousovic K, Konecny K, Mestecky J, Circulating immune complexes in IgA nephropathy consist of IgA1 with galactose-deficient hinge region and antiglycan antibodies, J. Clin. Invest 104 (1999) 73–81, 10.1172/JCI5535. 10393701 PMC408399

[11] Novak J, Raskova Kafkova L, Suzuki H, Tomana M, Matousovic K, Brown R, Hall S, Sanders JT, Eison TM, Moldoveanu Z, Novak L, Novak Z, Mayne R, Julian BA, Mestecky J, Wyatt RJ, IgA1 immune complexes from pediatric patients with IgA nephropathy activate cultured human mesangial cells, Nephrol. Dial. Transplant 26 (2011) 3451–3457, 10.1093/ndt/gfr448. 21828345 PMC3203630

[12] Takahashi K, Wall SB, Suzuki H, Smith AD, Hall S, Poulsen K, Kilian M, Mobley JA, Julian BA, Mestecky J, Novak J, Renfrow MB, Clustered *O*-glycans of IgA1: defining macro- and microheterogeneity by use of electron capture/transfer dissociation, Mol. Cell. Proteomics 9 (2010) 2545–2557, 10.1074/mcp.M110.001834. 20823119 PMC2984237

[13] Iwasaki H, Zhang Y, Tachibana K, Gotoh M, Kikuchi N, Kwon YD, Togayachi A, Kudo T, Kubota T, Narimatsu H, Initiation of O-glycan synthesis in IgA1 hinge region is determined by a single enzyme, UDP-N-acetyl-alpha-D-galactosamine:polypeptide N-acetylgalactosaminyltransferase 2, J. Biol. Chem 278 (2003) 5613–5621, 10.1074/jbc.M211097200. 12438318

[14] Reily C, Stewart TJ, Renfrow MB, Novak J, Glycosylation in health and disease, Nat. Rev. Nephrol 15 (2019) 346–366, 10.1038/s41581-019-0129-4. 30858582 PMC6590709

[15] Ju T, Brewer K, D’Souza A, Cummings RD, Canfield WM, Cloning and expression of human core 1 beta1,3-galactosyltransferase, J. Biol. Chem 277 (2002) 178–186, 10.1074/jbc.M109060200. 11677243

[16] Suzuki H, Moldoveanu Z, Hall S, Brown R, Vu HL, Novak L, Julian BA, Tomana M, Wyatt RJ, Edberg JC, Alarcon GS, Kimberly RP, Tomino Y, Mestecky J, Novak J, IgA1-secreting cell lines from patients with IgA nephropathy produce aberrantly glycosylated IgA1, J. Clin. Invest 118 (2008) 629–639, 10.1172/JCI33189. 18172551 PMC2157566

[17] Kiryluk K, Li Y, Moldoveanu Z, Suzuki H, Reily C, Hou P, Xie J, Mladkova N, Prakash S, Fischman C, Shapiro S, LeDesma RA, Bradbury D, Ionita-Laza I, Eitner F, Rauen T, Maillard N, Berthoux F, Floege J, Chen N, Zhang H, Scolari F, Wyatt RJ, Julian BA, Gharavi AG, Novak J, GWAS for serum galactose-deficient IgA1 implicates critical genes of the O-glycosylation pathway, PLoS Genet. 13 (2017), e1006609, 10.1371/journal.pgen.1006609. 28187132 PMC5328405

[18] Gale DP, Molyneux K, Wimbury D, Higgins P, Levine AP, Caplin B, Ferlin A, Yin P, Nelson CP, Stanescu H, Samani NJ, Kleta R, Yu X, Barratt J, Galactosylation of IgA1 is associated with common variation in C1GALT1, J. Am. Soc. Nephrol 28 (2017) 2158–2166, 10.1681/ASN.2016091043. 28209808 PMC5491291

[19] Suzuki H, Raska M, Yamada K, Moldoveanu Z, Julian BA, Wyatt RJ, Tomino Y, Gharavi AG, Novak J, Cytokines alter IgA1 *O*-glycosylation by dysregulating C1GalT1 and ST6GalNAc-II enzymes, J. Biol. Chem 289 (2014) 5330–5339, 10.1074/jbc.M113.512277. 24398680 PMC3931088

[20] Yamada K, Huang ZQ, Raska M, Reily C, Anderson JC, Suzuki H, Ueda H, Moldoveanu Z, Kiryluk K, Suzuki Y, Wyatt RJ, Tomino Y, Gharavi AG, Weinmann A, Julian BA, Willey CD, Novak J, Inhibition of STAT3 signaling reduces IgA1 autoantigen production in IgA nephropathy, Kidney Int Rep 2 (2017) 1194–1207, 10.1016/j.ekir.2017.07.002. 29270528 PMC5733772

[21] Yamada K, Huang Z-Q, Raska M, Reily CR, Aderson J, Suzuki H, Kiryluk K, Gharavi AG, Julian BA, Willey CD, Novak J, LIF signaling enhances production of galactose-deficient IgA1 in IgA nephropathy, Kidney Dis. 6 (2020) 168–180. 10.1159/000505748 PMC7265702 32523959

[22] Medrano AS, Muijsemberg A, Wimbury D, Martin M, Jatem E, Gonzalez J, Colas-Campas L, Garcia-Carrasco A, Martinez C, Barratt J, Relationship between immunoglobulin A1 lectin-binding specificities, mesangial C4d deposits and clinical phenotypes in immunoglobulin A nephropathy, Nephrol. Dial. Transplant 37 (2022) 318–325, 10.1093/ndt/gfaa356. 33315098

[23] Moore JS, Kulhavy R, Tomana M, Moldoveanu Z, Suzuki H, Brown R, Hall S, Kilian M, Poulsen K, Mestecky J, Julian BA, Novak J, Reactivities of *N*-acetylgalactosamine-specific lectins with human IgA1 proteins, Mol. Immunol 44 (2007) 2598–2604, 10.1016/j.molimm.2006.12.011. 17275907 PMC2788496

[24] Reily C, Rizk DV, Julian BA, Novak J, Assay for galactose-deficient IgA1 enables mechanistic studies with primary cells from IgA nephropathy patients, Biotechniques 65 (2018) 71–77, 10.2144/btn-2018-0042. 30091383 PMC6152805

[25] Reily C, Xu N, Crossman DK, Assigning immunoglobulin class from single-cell transcriptomes in IgA1-secreting versus membrane subpopulations, Biotechniques 70 (2021) 89–99, 10.2144/btn-2020-0044. 33307788 PMC7983040

[26] Wang YN, Zhou XJ, Chen P, Yu GZ, Zhang X, Hou P, Liu LJ, Shi SF, Lv JC, Zhang H, Interaction between G ALNT12 and C1GALT1 associates with galactose-deficient IgA1 and IgA nephropathy, J. Am. Soc. Nephrol 32 (2021) 545–552, 10.1681/ASN.2020060823. 33593824 PMC7920185

[27] Kiryluk K, Moldoveanu Z, Sanders JT, Eison TM, Suzuki H, Julian BA, Novak J, Gharavi AG, Wyatt RJ, Aberrant glycosylation of IgA1 is inherited in both pediatric IgA nephropathy and Henoch-Schőnlein purpura nephritis, Kidney Int. 80 (2011) 79–87, 10.1038/ki.2011.16. 21326171 PMC3641561

[28] Kiryluk K, Li Y, Scolari F, Sanna-Cherchi S, Choi M, Verbitsky M, Fasel D, Lata S, Prakash S, Shapiro S, Fischman C, Snyder HJ, Appel G, Izzi C, Viola BF, Dallera N, Del Vecchio L, Barlassina C, Salvi E, Bertinetto FE, Amoroso A, Savoldi S, Rocchietti M, Amore A, Peruzzi L, Coppo R, Salvadori M, Ravani P, Magistroni R, Ghiggeri GM, Caridi G, Bodria M, Lugani F, Allegri L, Delsante M, Maiorana M, Magnano A, Frasca G, Boer E, Boscutti G, Ponticelli C, Mignani R, Marcantoni C, Di Landro D, Santoro D, Pani A, Polci R, Feriozzi S, Chicca S, Galliani M, Gigante M, Gesualdo L, Zamboli P, Battaglia GG, Garozzo M, Maixnerova D, Tesar V, Eitner F, Rauen T, Floege J, Kovacs T, Nagy J, Mucha K, Paczek L, Zaniew M, Mizerska-Wasiak M, Roszkowska-Blaim M, Pawlaczyk K, Gale D, Barratt J, Thibaudin L, Berthoux F, Canaud G, Boland A, Metzger M, Panzer U, Suzuki H, Goto S, Narita I, Caliskan Y, Xie J, Hou P, Chen N, Zhang H, Wyatt RJ, Novak J, Julian BA, Feehally J, Stengel B, Cusi D, Lifton RP, Gharavi AG, Discovery of new risk loci for IgA nephropathy implicates genes involved in immunity against intestinal pathogens, Nat. Genet 46 (2014) 1187–1196, 10.1038/ng.3118. 25305756 PMC4213311

[29] Valentijn RM, Radl J, Haaijman JJ, Vermeer BJ, Weening JJ, Kauffmann RH, Daha MR, van Es LA, Circulating and mesangial secretory component-binding IgA-1 in primary IgA nephropathy, Kidney Int. 26 (1984) 760–766, 10.1038/ki.1984.213. 6441067

