# Supplementary material for: Phosphatase control of cytokine-mediated overproduction of galactose-deficient IgA1, the main autoantigen in IgA nephropathy
Source: J Autoimmun. Author manuscript; Available in PMC 2022 Nov 20. (PMC9675727; doi:10.1016/j.jaut.2022.102883)
Supplement: Supplemental.Data.Reily.IgAN.phosphatase.2022 [file NIHMS1839012-supplement-Supplemental_Data_Reily_IgAN_phosphatase_2022.pdf]

Supplemental Figures and Tables

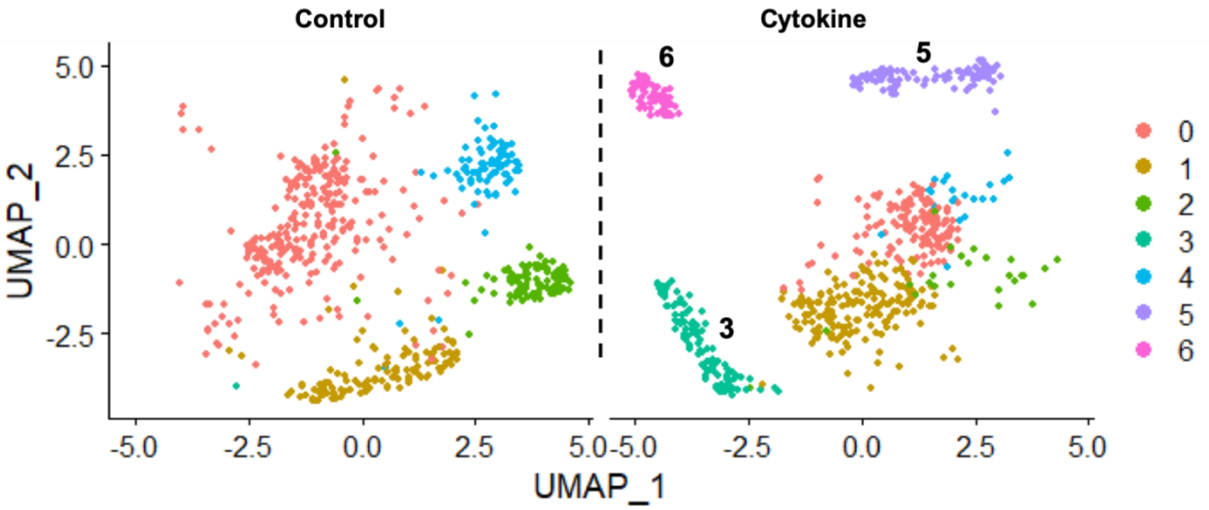

S.Fig.1: Single-cell transcriptomic UMAP of healthy control immortalized B cells before and after cytokine stimulation: Immortalized B cells from HC donors (N=4) were stimulated with cytokines for 20 min, and subjected to single-cell transcriptomic analysis. Seurat v4.0 was used to normalize data, *IGHA1s* subpopulations were grouped, and UMAP was used to find common and distinct populations between unstimulated (control) and stimulated (cytokine) samples positive for IgA1-secretion. The numbers (3,5,6) listed on the graph indicate unique populations for HC after cytokine stimulation.

	C1GALT1		*C1GALT1C1*		*GALNT12*		*SOCS1*		*SOCS3*		*PTPN2*		*PTPN6*		*PTPN11*	
**Group**	**Log2FC**	**p-adj**	**Log2FC**	**p-adj**	**Log2FC**	**p-adj**	**Log2FC**	**p-adj**	**Log2FC**	**p-adj**	**Log2FC**	**p-adj**	**Log2FC**	**p-adj**	**Log2FC**	**p-adj**
**0**	0.57	1	ND	ND	1.16	3.16E-23	ND	ND	ND	ND	ND	ND	ND	ND	ND	ND
**1**	ND	ND	0.33	1.67E-05	ND	ND	0.52	2.25E-09	1.04	6.64E-25	ND	ND	ND	ND	0.30	1
**2**	ND	ND	0.32	6.15E-08	0.41	0.00	ND	ND	-0.69	0.11	ND	ND	0.48	1	ND	ND
**3**	ND	ND	-0.47	0.23	-1.50	1.30E-17	ND	ND	ND	ND	ND	ND	ND	ND	ND	ND
**4**	-0.42	1	ND	ND	ND	ND	-0.91	5.92E-06	ND	ND	-0.54	1	-0.39	1	-0.33	1
**5**	ND	ND	-0.60	0.35	-1.50	4.74E-16	-0.95	1	-0.95	2.02E-07	ND	ND	ND	ND	ND	ND
**6**	ND	ND	-1.14	1	-1.47	1.50E-09	ND	ND	-0.50	0.12	ND	ND	-0.47	1	-1.28	3.4E-4

S.Table.1: Differential regulation of glycosyltransferases and phosphatases in *IGHA1s* subpopulations from HC donors: Immortalized B cells from HC donors (N=4) were stimulated with cytokines for 20 min, and subjected to single-cell transcriptomic analysis. Seurat v4.0 was used to normalize data, *IGHA1s* subpopulations were grouped, and UMAP was used to find common and distinct populations between unstimulated (control) and stimulated (cytokine) samples positive for IgA1-secretion. Select glycosyltransferases and phosphatases are listed. ND = genes with no differential expression from other groups.

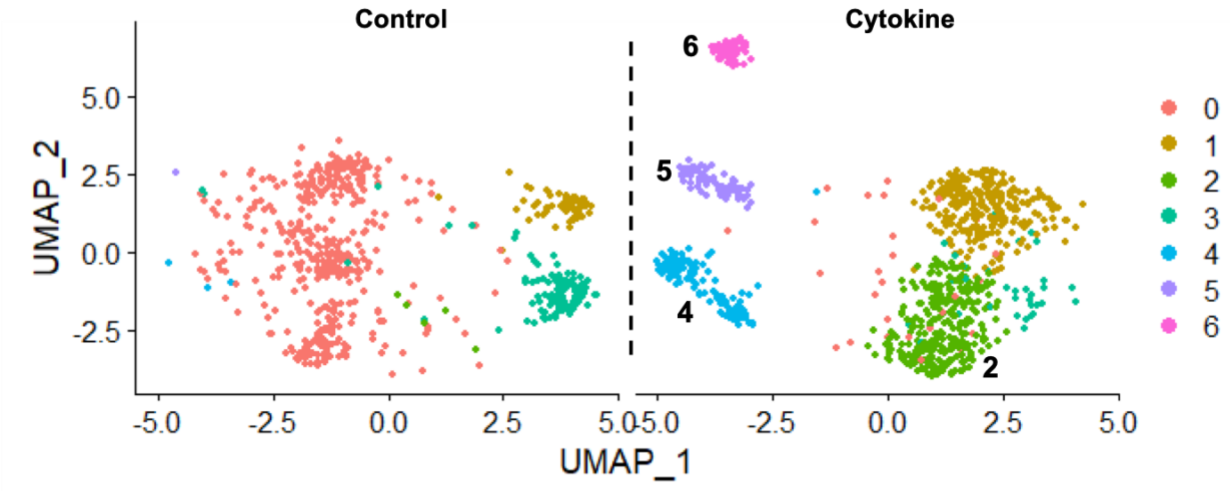

S.Fig.2: Single-cell transcriptomic UMAP of IgAN immortalized B cells before and after cytokine stimulation: Immortalized B cells from IgAN donors (N=4) were stimulated with cytokines for 20 min, and subjected to single-cell transcriptomic analysis. Seurat v4.0 was used to normalize data, *IGHA1s* subpopulations were grouped, and UMAP was used to find common and distinct populations between unstimulated (control) and stimulated (cytokine) samples positive for IgA1-secretion. The numbers listed (2, 4, 5, 6) on the graph indicate unique populations for IgAN after cytokine stimulation.

	C1GALT1		*C1GALT1C1*		*SOCS1*		*SOCS3*		*PTPN2*		*PTPN6*		*PTPN11*		*GALNT12*	
**Group**	**Log2FC**	**p-adj**	**Log2FC**	**p-adj**	**Log2FC**	**p-adj**	**Log2FC**	**p-adj**	**Log2FC**	**p-adj**	**Log2FC**	**p-adj**	**Log2FC**	**p-adj**	**Log2FC**	**p-adj**
**0**	ND	ND	0.57	5.36E-23	ND	ND	ND	ND	ND	ND	ND	ND	ND	ND	0.49	5.74E-14
**1**	ND	ND	-0.28	0.04	0.33	2.87E-05	ND	ND	ND	ND	ND	ND	ND	ND	ND	ND
**2**	0.68	1.08E-06	ND	ND	ND	ND	0.37	7.04E-08	ND	ND	0.57	9.13E-25	0.29	0.03	-0.35	7.84E-06
**3**	ND	ND	-0.28	0.78	-0.39	1	ND	ND	-0.43	0.02	ND	ND	-0.67	0.01	-0.34	0.0377758
**4**	-0.50	6.04E-12	ND	ND	ND	ND	0.27	1	ND	ND	-0.29	2.98E-05	ND	ND	ND	ND
**5**	ND	ND	ND	ND	ND	ND	-1.24	0.01	ND	ND	-0.58	1	-0.25	2.34E-10	ND	ND
**6**	-0.88	7.81E-05	ND	ND	-1.04	0.67	-1.07	1	-0.99	6.11E-06	-0.27	1	ND	ND	ND	ND

S.Table.2: Differential regulation of glycosyltransferases and phosphatases in *IGHA1s* subpopulations from IgAN donors: Immortalized B cells from IgAN donors (N=4) were stimulated with cytokines for 20 min, and subjected to single-cell transcriptomic analysis. Seurat v4.0 was used to normalize data, *IGHA1s* subpopulations were grouped, and UMAP was used to find common and distinct populations between unstimulated (control) and stimulated (cytokine) samples positive for IgA1-secretion. Select glycosyltransferases and phosphatases are listed. ND = genes with no differential expression from other groups.

Select Genes after Cytokine Stimulation								
	PTPN2		***PTPN6***		***PTPN11***		***NFKBIA***	
**Group**	**LFC**	**p-adj**	**LFC**	**p-adj**	**LFC**	**p-adj**	**LFC**	**p-adj**
**0**	ND	ND	ND	ND	0.61884	9.50E-25	0.497046	1.06E-14
**1**	-0.34848	1	0.436418	1.07E-17	ND	ND	-0.4024	4.52E-05
**2**	ND	ND	-0.31246	0.000552	-0.52951	1	ND	ND
**3**	-0.25855	1	-0.60006	0.00792	-0.74387	2.98E-19	ND	ND
**4**	ND	ND	ND	ND	ND	ND	ND	ND
**5**	ND	ND	-0.29677	3.53E-07	ND	ND	ND	ND
**6**	-1.04084	3.64E-06	-0.2942	1	-0.71197	0.154679	-1.36775	4.96E-10

S.Table.3: Differential regulation of select genes in *IGHA1s* subpopulations after cytokine stimulation: Immortalized B cells from IgAN donors (N=4) were stimulated with cytokines for 20 m, and subjected to single-cell transcriptomic analysis. Seurat v4.0 was used to normalize data, *IGHA1s* subpopulations were grouped, and UMAP was used to find common and distinct populations between healthy control and IgAN samples positive for IgA1-secretion. Select genes are listed. ND = genes not determined to be different in that group. *PTPN2/6/11 (protein tyrosine phosphatase non-receptor type 2/6/11), NFKBIA (NF-kappa-B inhibitor A).*
